# Coupling and uncoupling of triglyceride and beta-carotene production by *Dunaliella salina* under nitrogen limitation and starvation

**DOI:** 10.1186/s13068-017-0713-4

**Published:** 2017-01-31

**Authors:** Hubert Bonnefond, Nina Moelants, Amélie Talec, Patrick Mayzaud, Olivier Bernard, Antoine Sciandra

**Affiliations:** 1 UPMC Univ Paris 06, INSU-CNRS, Laboratoire d’Océanographie de Villefranche, Sorbonne Universités, 181 Chemin du Lazaret, 06230 Villefranche-sur-mer, France; 2 INRIA, BIOCORE, Université Nice Côte d’Azur, 06902 Sophia Antipolis, France

**Keywords:** *Dunaliella salina*, Triglycerides, Beta-carotene separation, Nitrogen starvation/limitation, Droop model

## Abstract

**Background:**

Nitrogen starvation and limitation are known to induce important physiological changes especially in lipid metabolism of microalgae (triglycerides, membrane lipids, beta-carotene, etc.). Although little information is available for *Dunaliella salina*, it is a promising microalga for biofuel production and biotechnological applications due to its ability to accumulate lipid together with beta-carotene.

**Results:**

Batch and chemostat experiments with various degrees of nitrogen limitation, ranging from starvation to nitrogen-replete conditions, were carried out to study carbon storage dynamics (total carbon, lipids, and beta-carotene) in steady state cultures of *D. salina*. A new protocol was developed in order to manage the very high beta-carotene concentrations and to more accurately separate and quantify beta-carotene and triglycerides by chromatography. Biomass evolution was appropriately described by the Droop model on the basis of the nitrogen quota dynamics.

**Conclusions:**

Triglycerides and beta-carotene were both strongly anti-correlated with nitrogen quota highlighting their carbon sink function in nitrogen depletion conditions. Moreover, these two valuable molecules were correlated each other for nitrogen replete conditions or moderated nitrogen limitations (N:C ratio higher than 0.04). Under nitrogen starvation, i.e., for very low N:C ratio, the dynamic revealed, for the first time, uncoupled part (higher triglyceride accumulation than beta-carotene), possibly because of shortage in key proteins involved in the stabilization of lipid droplets. This study motivates the accurate control of the microalgal nitrogen quota in order to optimize lipid productivity.

## Background

In the context of climate changes and increasing energy requirements, photosynthetic microorganisms transforming a large fraction of incorporated CO_2_ into storage carbon molecules, especially triglycerides or carbohydrates have gained interest. Exploring microalgae diversity brings new possibilities to achieve high bioenergy production yields with a reduced environmental impact [1]. Among the species of interest for biofuel production, *Dunaliella salina* is an halotolerant green algae able to grow in extreme saline environments (up to 350 g L^−1^ NaCl), limiting therefore contaminations by competitors and predators [2, 3].

*Dunaliella salina* can produce high amounts of total lipids, depending on the growth conditions (6.0–25.0% dw; [4]). Information on triglyceride content is rarely presented, probably because of the strong interaction with beta-carotene in classical measurement protocols. Indeed, *D. salina* is one of the living organisms with the highest content in beta-carotene, reaching 10% of the dry weight under stress conditions. Beta-carotene belongs to carotenoid molecules which constitute a class of natural terpenoid pigments derived from a 40-carbon polyene chain. This backbone is complemented by aromatic cycles and oxygenated functional groups [5]. The nature of the functional groups of carotenoids affects polarity, chemical properties, and oxidation degree. The main beta-carotene characteristics are: very low polarity and high number of double bonds. These characteristics are responsible for powerful antioxidant properties. Beta-carotene is produced in thylakoids as a photosynthetic product and accumulated into lipid droplets located in the inter-thylakoid spaces [6], in the chloroplast [7], and/or in the cytoplasm [8, 9]. Lipid droplets are surrounded by a stabilizing monolayer of phospholipids and specific proteins and form connected organelles [10]. Pathways of beta-carotene biosynthesis are now understood but control mechanisms remain unclear [11]. Rare studies have highlighted a probable relationship between lipids and beta-carotene accumulation in this species [12, 13], while triglyceride synthesis may trigger beta-carotene production. This link remains poorly understood and documented.

Overproduction of total lipids and beta-carotene after nitrogen starvation (i.e., in conditions of unbalanced growth in response to total lack of nitrogen [14]) is well documented for *D. salina* [13], since most of the physiological studies have been carried out in batch conditions. However, the physiological state achieved in chemostat is significantly different. In such continuous culture mode, cells acclimate to nitrogen limitation with a reduced, but balanced, growth rate [14]. A key difference between these two nitrogen stresses is that steady state cannot be reached under the unbalanced starvation conditions (unless cell death is considered as the final state). No information is available concerning triglycerides and beta-carotene production under nitrogen limitation while it is known to induce very different responses compared to nitrogen starvation [15].

This study focuses on the lipid storage strategy in *D. salina*, comparing the responses for nitrogen limitation and starvation. To address this question, we used chemostat experiments which provide a sound framework for studying the effect of various nutrient limitation rates on steady-state cultures [16]. We also developed a new methodological protocol by column chromatography to accurately co-analyze the high beta-carotene and lipid contents found in our samples. We show that beta-carotene accumulation is strongly related to nitrogen cell quota, and highlight a coupling between triglyceride and beta-carotene storage.

## Methods

### Culturing system

*Dunaliella salina* (CCAP 18/19) was cultivated in two duplicate 5 L water-jacketed cylindrical photobioreactors used in chemostat mode. Before starting continuous mode and nitrogen limitation treatment, microalgae were grown in the photobioreactors for 15 days in replete conditions in order to stabilize biomass, growth rate, and allow algae to acclimate to culturing conditions.

**Fig. 1:**
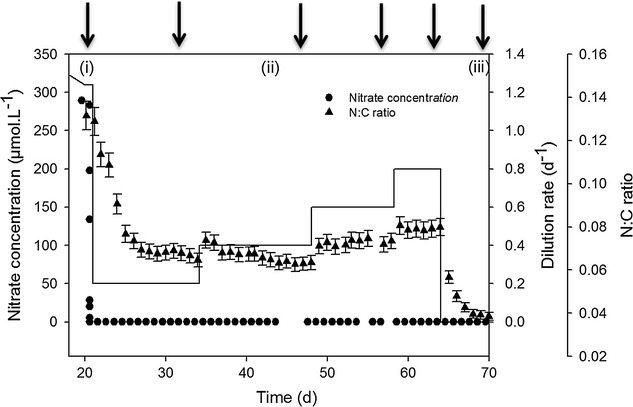
Average dilution rate, nitrate concentration, and N:C ratio in microalgae. *Black arrows* represent sampling for lipid measurements at culture steady state. Cultures were successively N-replete (*i*), N-limited (*ii*), and N-starved (*iii*)

The enrichment medium was prepared in 20 L tanks (Nalgen) filled with 3 weeks of matured sea water pumped at the surface of the Villefranche bay (France), filtered through 0.1 µm Millipore and autoclaved at 110 °C for 20 min. After cooling, f/2 medium [17] was added. NO_3_^−^ was lowered to obtain a final concentration (*s*_0_) of 260 µmol L^−1^ in the inflowing medium. This concentration allowed the culture biomass to stabilize at a level where self-shading was low and cell density sufficient to obtain accurate biochemical analyses from small volume samples (between 2 and 5.5 × 10^8^ cell L^−1^ depending on the dilution rate applied). Renewing medium was added into photobioreactors by peristaltic pumps (Gilson) after online filtration with 0.22 µm sterile filters (SpiralCap, Gelman). Each day, the dilution rate (*D*) was measured by weighting the input flow with a precision balance and adjusted to the chosen value if necessary. The dilution rate was modified for each new experiment phase, in order to reach different growth rates in the chemostat, associated to different intracellular cell quotas. (a) N replete conditions were obtained by setting *D* to the maximum growth rate measured during the pre-cultivation phase (1.1 d^−1^). (b) Different levels of N limitation for different growth rates and physiological states were obtained by setting *D* at 0.2, 0.4, 0.6, and 0.8 d^−1^ successively. (c) N starved cultures were obtained in batch mode (*D* = 0 d^−1^, Fig. 1).

### Physical parameters

Photobioreactors were connected to a cryostat (Lauda RE 415G) maintaining a constant temperature of 25 °C. Light was provided by two arrays of six 50 cm fluorescent tubes (Dulux^®^1, 2G11, 55 W/12-950, Lumilux de lux, daylight, OsramSylvania, Danvers, MA, USA). Photosynthetic active radiation (PAR) was continuously recorded with a spherical collector (QSL-100, Biospherical Instruments, San Diego, CA, USA) placed between the two duplicate photobioreactors, and was adjusted to 400 µmol quanta m^−2^ s^−1^. Continuously bubbled air was passed through active charcoal and 0.1 µm Whatman filter. pH was maintained at 8.3 by computer-controlled micro-injections of CO_2_ in the bubbled air [18].

### Nitrate measurement

Nitrate and nitrite were daily measured with a Technicon Auto-analyzer coupled to an automated sampling and filtering device as described by [19].

### Cell concentration and total biovolume

Cell density (cell L_medium_^−1^) and size spectra were measured every 3 h in photobioreactors using a computer-controlled automata connected to an optical particle counter (HIAC/Royco; Pacific Scientific Instruments, Grants Pass, OR, USA). Variability between triplicate measurements was routinely <5%. Total biovolume (L_cell_ L_medium_^−1^) was calculated from the size spectra assuming that cells were spherical.

### Particulate C and N

Triplicate sampling was performed once a day (Fig. 1). 12.64 mL of culture were filtered onto glass-fiber filters (Watman GF/C, Maidstone, UK) precombusted at 450 °C for 12 h. Samples were kept at 60 °C before analysis with a CHN analyzer (2400 Series II CHNS/O, Perkin Elmer, Norwalk, CT, USA). Continuous functions of Savitzky Golay filter [20] were fitted to carbon data series (µgC mL^−1^), and the derivate was used to compute the net carbon specific growth rates *µ* (µgC µgC^−1^ d^−1^) according to the following equation:

1 $$\mu = \frac{1}{{X_{{t_{2} }} }}\left( {\frac{{X_{{t_{2} }} - X_{{t_{1} }} }}{{\left( {t_{2} - t_{1} } \right)}} + D \cdot X_{{t_{2} }} } \right),$$

where *X* is the particulate carbon concentration or carbon biomass concentration (µgC mL^−1^) at time *t*_1_ and *t*_2_, respectively.

### Chemostat model

In a continuous culture, the time variations of microalgae biomass *X* and limiting nutrient *s* can be described by the Droop model (Droop 1983):

2 $$\frac{{{\text{d}}X}}{{{\text{d}}t}} = \mu X - DX$$

3 $$\frac{{{\text{d}}s}}{{{\text{d}}t}} = Ds_{0} - Ds - \rho \left( s \right)X$$

with *µ* the growth rate, *D* the dilution rate, *s*_o_ the concentration of the limiting nutrient in the enrichment medium, and *ρ*(*s*) the uptake rate of the limiting nutrient per unit of *X*, which can be represented by the Michaelis–Menten equation:

4 $$\rho \left( s \right) = \rho_{\text{m}} \frac{s}{{s + k_{\text{s}} }}$$

with *ρ*_m_ the maximum uptake rate and *k*_s_ the half saturation constant that can be determined experimentally.

Droop [16, 21] showed that the growth rate *µ* is related to the internal quota (*q*) of the limiting nutrient (originally vitamin B_12_), according to the following expression:

5 $$\mu \left( q \right) = \bar{\mu }\left( {1 - \frac{{q_{0} }}{q}} \right).$$

In our experiment, *q* was the amount of nitrogen per carbon unit (i.e., the N:C ratio), *q*_0_ the minimum value of *q* below which growth was not possible, and $\bar{\mu }$ the theoretical maximum growth rate obtained when *q* is assumed to be infinite.

The equation describing the time variation of *q* is:

6 $$\frac{{{\text{d}}q}}{{{\text{d}}t}} = \rho \left( s \right) - \mu \left( q \right) q.$$

At steady state, i.e., when *µ* = *D*, see (1), it was demonstrated [22] that the microalgae biomass, X* (µgC L^−1^), is a function of the dilution rate *D* and *s*_o_ according to the following expression:

7 $$X^{*} = \frac{{\left( {\bar{\mu } - D} \right)\left( {\bar{\mu }\rho_{m} s_{0} - D\rho_{m} s_{0} - \bar{\mu }q_{0} k_{s} D - \bar{\mu }q_{0} Ds_{0} } \right)}}{{\bar{\mu }q_{0} \left( {\bar{\mu }\rho_{m} - \bar{\mu }q_{0} D - D\rho_{m} } \right)}}.$$

### Lipid sampling and extraction

For each dilution rate tested, the lipids were sampled once the cultures reached their presumed state of equilibrium (Fig. 1). 320 mL of culture were centrifuged (JOUAN G 412) 10 min at 490*g*, and the pellets were stored at −80 °C to prevent enzymatic degradation of lipids. Lipid extraction was derived from Bligh and Dyer’s method [23] as described by [24] after a mechanical homogenization leading to cell disruption (*D. salina* does not have any cell wall). Total lipids were dosed gravimetrically, and then stored until further analysis at −80 °C under N_2_ atmosphere to avoid oxidation.

### Beta-carotene separation

A method to separate beta-carotene from others lipids was specifically developed to improve analyses. Micro-columns (Grace Davison, silica 500 mg, 8 mL) were activated and cleaned. The following sequence of solvents was used: one column volume of hexane with 1.6% of diethyl-ether to elute beta-carotene (Fraction A); 5 and 3 column volumes of chloroform:methanol 1:1, and methanol, respectively, to elute other lipids (fraction B’). The different fractions obtained were dried under N_2_ and stored at −80 °C under N_2_ atmosphere, before further analysis. The beta-carotene fraction was determined gravimetrically.

### Lipid class determination

After the beta-carotene separation, lipid classes were quantified on the fraction B’ without beta-carotene using thin layer chromatography (TLC) coupled with FID (flame photometric detection) in triplicate as described [24]. A known mass of lipids was automatically spotted (SES A4100 Autospotter) onto silica coat rods (Chromarod-SIII), and then developed with two successive solvent systems (80 mL hexane/20 mL benzene/1 mL formic acid and 97 mL hexane/3 mL ether/1.5 mL formic acid). Lipid class determination was performed with a Iatroscan (Iatroscan New MK 5 from Iatron and integrated using the software: Chromstar from SES Analysesysteme) [25]. The variability between triplicate measurements of triglycerides was routinely lower than 10% and less than 3% for polar lipids.

### Statistical analysis

For each limitation rate tested, each value represents the average of *n* ≥ 6 ± SD, measured on the two independent highly instrumented photobioreactors. Tests were considered significant for *p* values <0.05.

## Results

### A new beta-carotene separation protocol

To develop a reliable protocol to separate beta-carotene from other lipids in *D. salina*, a step-by-step analysis of the column chromatography process was performed following the methodology presented in Table 1. Four fractions were recovered and weighed: two majors (A and D) and two minors (B and C). This separation protocol was very reproducible. The mass coefficient of variation was lower than 5% (*n* = 9) and included all the steps from biomass recovery by centrifugation to column separation.

**Table 1:** Sequence of solvents used in the method of separation of total lipids and beta-carotene

Spotting of the sample	0.5 mL of hexane, 1.6% of diethyl-ether
Fraction A	One column volume of hexane, 1.6% of diethyl-ether
Fraction B	One column volume of hexane, 1.6% of diethyl-ether
Fraction C	One column volume of hexane, 1.6% of diethyl-ether
Fraction D	Five column volume of chloroform/methanol (1:1) Three column volume of methanol

Each fraction was then analyzed by thin-layer chromatography to validate the method resolution

**Fig. 2:**
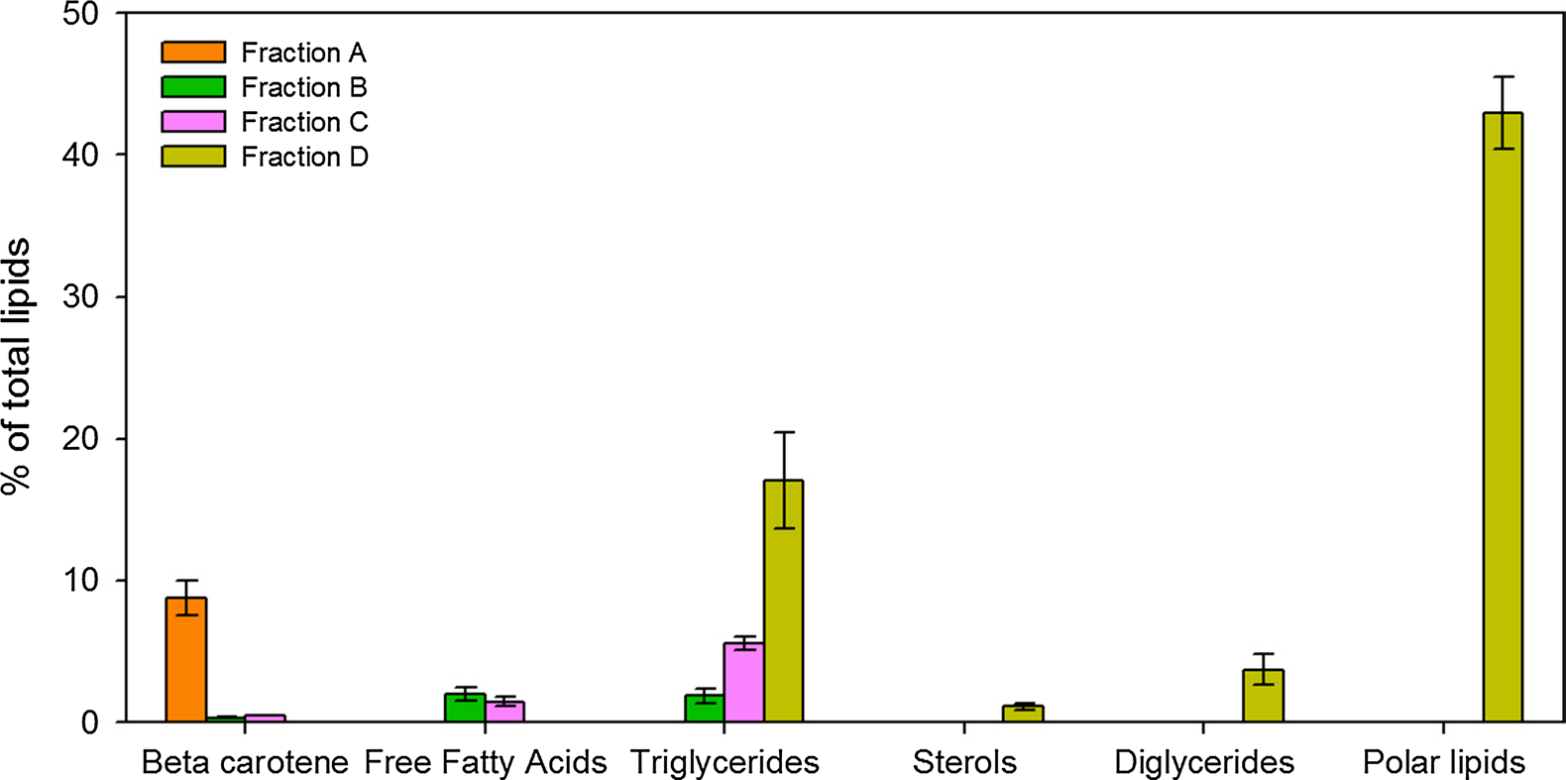
Characterization of the degree of separation of beta-carotene from other lipid classes using thin-layer chromatography coupled with flame ionisation detector. The lipid class composition of each fraction obtained during the separation protocol is represented as a fraction of total lipids

The four fractions were then analyzed in triplicates by thin-layer chromatography (see “Methods” and [24]) to determine their lipid class compositions (Fig. 2). Due to the very low polarity of the solvent used to recover fraction A (hexane + 1.6% diethyl-ether), the orange lipids weighted at the end of this step could only be carotene hydrocarbon (alpha or beta-carotene) [26]. As *D. salina* produces mainly beta-carotene, the recovered fraction was composed of more than 75% beta-carotene [27]. Nevertheless, after thin-layer chromatography, six colored molecules were identified in fraction A, corresponding visually to yellow/orange spots (data not shown). The most abundant and situated in the more non-polar position was beta-carotene. The other molecules were located in more polar positions. Beta-carotene is very sensitive to oxidation [26, 28], and during separation, it is exposed to oxidative conditions such as oxygenated solvents, di-oxygen, light, and temperature [29]. The beta-carotene oxidation products (carotene epoxides) present higher polarity than beta-carotene since they contain oxygen [5]. Keeping generally their yellow/orange color, except in cases of very strong oxidation, carotene epoxides were eluted slowly during the thin-layer chromatography [26], which likely explain the different yellow spots in more polar positions than beta-carotene. Alternatively, it could also result from different conformations of the beta-carotene which present different polarities (cis and trans; [26, 30]) or different carotene hydrocarbons in very small proportion (<1%; alpha, delta or gamma carotenes, phytoene, etc.). As a conclusion, carotenes were totally separated from other lipids during this step and quantified gravimetrically, even if this process probably lead to carotene oxidation (which did not alter the mass quantification).

Fractions B and C represented lower mass fractions (<8%), and were mainly composed of triglycerides and free fatty acids (Fig. 2). Beta-carotene residues (about 8%) were found (orange spots in non-polar position) in these fractions. Fraction D was composed of triglycerides, vegetal–sterols, diglycerides, and polar lipids such as phospholipids and glycolipids, or polar pigments such as chlorophyll a or xanthophylls (Fig. 2). This protocol was non-oxidative for lipids, as confirmed by the low content of free fatty acids (oxidative marker) accounting for <2% of total lipids. Note that for routine measurements, the protocol was reduced to two steps, the first one to recover beta-carotene (one volume of hexane, 1.6% diethyl-ether) and the second one to recover the remaining lipids (five and three column volumes of chloroform:methanol 1:1 and methanol, respectively).

### Steady states under nitrogen limitation

**Fig. 3:**
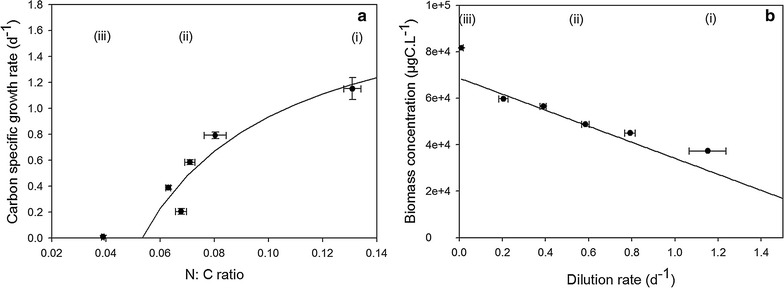
**a** Carbon-specific growth rate as a function of the nitrogen quota (N:C) measured at steady state for different dilution rates and fitted to the Droop equation (Eq. 5; *black line*). **b** Steady-state algal carbon concentrations measured (*black symbols*) and calculated with Eq. 7 (*black line*). Three phases can be distinguished (*i*) N-replete, (*ii*) N limitation, and (*iii*) N starvation

The expected Droop relationship between growth rate and nitrogen quota measured at steady state [16] was verified (Fig. 3a, b). Parameters $\bar{\mu }$ and *q*_o_ were estimated (Table 2) by fitting Eq. (5) to these data. Note that the data obtained for *D* = 0 were not considered in the regression, as they were not acquired in continuous culture conditions. *ρ*_m_ was experimentally estimated, while the *k*_s_ value was taken from the literature [31]. The theoretical steady-state algal biomass as a function of the dilution rate was then computed according to Eq. (7).

**Table 2:** *D. salina* parameters for Droop and Michaelis–Menten equations

Parameters	Units	Value
$\bar{\mu }$	d^−1^	1.99
*q*_0_	µgN µgC^−1^	0.053
*ρ*_m_	µgN µgC^−1^ d^−1^	0.15
*k*_s_	µgN L^−1^	18

### Response of lipid composition to the nitrogen status

**Fig. 4:**
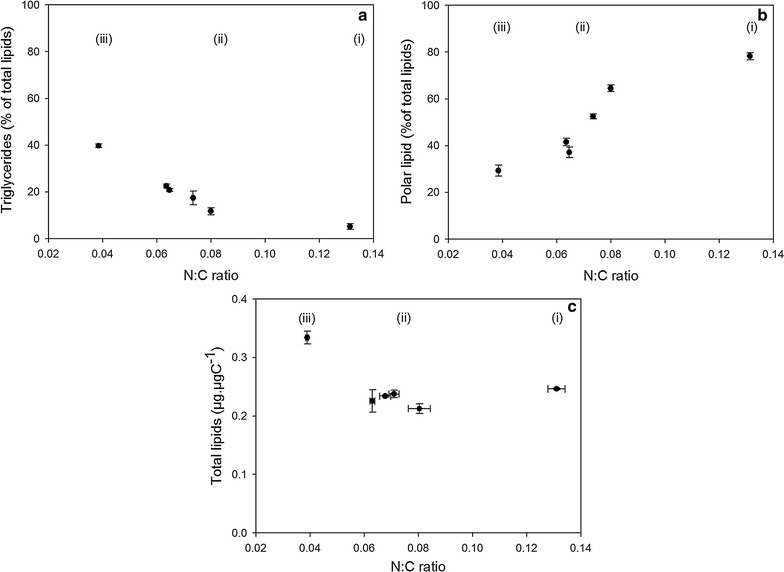
Responses of triglycerides (**a**), polar lipids (**b**), and total lipids (**c**) quotas to the nitrogen status (N:C). Three phases can be distinguished (*i*) N-replete, (*ii*) N limitation, and (*iii*) N starvation

Under nitrogen limitation and starvation, triglycerides were negatively correlated with the nitrogen quota (*R* ^2^ adj = 0.99; *n* = 10; statistic error lower than 5%). This correlation was less marked in nitrogen-replete conditions (high N:C ratio; Fig. 4a). Polar lipids were positively correlated with N:C ratio (Fig. 4b) and shifted from 0.12 ± 0.004 for N starvation to 0.24 ± 0.009 µg µgC^−1^ for N replete conditions. Total lipids remained constant, and were not related to nitrogen quota (0.23 ± 0.01 µg µgC^−1^). Nevertheless, under strong nitrogen starvation, i.e., when N:C decreased down to 0.04 g g^−1^, total lipids increased by 44.5% to reach 0.33 ± 0.02 µg µgC^−1^ (Fig. 4c).

### Beta-carotene versus triglyceride contents

**Fig. 5:**
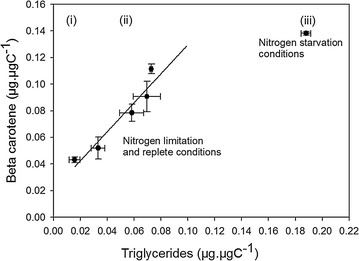
Beta-carotene content versus triglycerides quotas measured for different nitrogen statuses of *D. salina*. Three phases can be distinguished (*i*) N-replete, (*ii*) N limitation, and (*iii*) N starvation

A positive and significant (*R* ^2^ adj = 0.87; *n* = 14; statistic error lower than 5%) covariation of beta-carotene and triglyceride contents was observed in response to the rate of N limitation (Fig. 5). When condition became extreme, N starvation introduced a decoupling of triglycerides and beta-carotene.

## Discussion

### A new method able to discriminate lipid classes in very rich carotenoids content microalgae species

Our experiment shows that beta-carotene represented a significant proportion of total lipids (30%) in N-limited cells of *D. salina*. The chemical specificities of beta-carotene made it a potential source of errors for its accurate determination as well as for the determination of other lipid classes [5]. First, because of its low polarity, beta-carotene had the properties of a solvent and co-elutes other lipids during thin layer chromatography. Second, the oxidative products of beta-carotene with higher polarities are partially eluted together with other lipid classes. Third, the very viscous mixture of beta-carotene and other lipids introduces practical difficulties for chromatographic separation by blocking auto-spotter syringe during the lipid class analysis. These factors decreased both the sensitivity and the precision of the analyses. Several methodologies proposed to separate beta-carotene from other carotenoids [32, 33], but the accurate separation and co-determination of lipid classes (polar lipids, triglycerides, etc.) and beta-carotene remains rare [34, 35]. Moreover, beta-carotene and other lipid classes are usually quantified by independent methods (thin-layer chromatography and HPTLC or spectrophotometric approaches), which is time consuming [26].

Using preliminary results of Hirsch and Ahrens [36] and Carroll [37], we confirmed that the mix 1.6% of diethyl ether in hexane was optimal to separate beta-carotene from triolein standards. This new chromatographic separation method with silica micro-columns was then demonstrated to be tailor to the specific lipid mix of *D. salina*. The use of industrial micro-columns resulted in a better reproducibility, while minimizing the solvent and sample volumes (<15 mL and 3 mg, respectively). As oxidative products were found in the carotene fraction (fraction A), the protocol could be further improved by limiting light exposition, avoiding oxygenated solvents, and keeping temperature below 30 °C during solvent evaporation.

### Nitrogen status of microalgae during the experiment

Algal carbon and growth rates measured in our duplicate continuous cultures at steady state were accurately predicted by the Droop model. This shows that nitrogen limitation was effective and in accordance with chemostat theory at both the cell (Fig. 3a) and culture (Fig. 3b) levels. Consequently, growth was balanced for the different conditions tested, confirming that lipid status was analyzed in stationary metabolic activities. Despite this effective agreement between data and model, the growth rate measured diverged from the Droop predictions (Fig. 3a) for dilution rates lower than 0.2 d^−1^. In these conditions, the C:N ratio was higher than expected (Fig. 3b) suggesting that the steady state was not reached at the cellular level. Various authors have reported difficulties in observing the expected steady-state cell concentrations in continuous cultures maintained at low dilution rates [15, 16, 22], where supplementary limiting factors others than nitrogen can emerge (increased competition with bacteria, light and CO_2_ limitation, accumulation of deleterious bio-products…, etc.).

### Lipid accumulation under severe N-limited conditions

In starvation or for very high nitrogen limitations (very low dilution rates), the decrease in the nitrogen quota (N:C ratio) led to an increase in triglyceride content. This response is in agreement with the literature for other species and seems consistent for most of the eukaryote microalgae [15, 38–40]. To the best of our knowledge, such results were never reported before for *D. salina*. For this species, triglycerides content can reach 9% of the cell dry weight (assuming that carbon represents 50% of the dw), a value close to the 10% reported for *Dunaliella tertiolecta* by [40].

The total lipid content varied between 10 and 16% of dry weight similar to the range indicated by [4] (6–25% of dw). Nevertheless, the effects of nitrogen limitation or starvation on total lipids have been poorly investigated in *D. salina*, and the observations reported in the literature are contradictory. A 44.5% increase in total lipids was observed in N-starved conditions (Fig. 4c), whereas no significant change was detected by [13, 41] in similar conditions. In these studies, lipids were measured right after exhaustion of nitrogen in the culture (at the end of the log phase and 3 days after, respectively), whereas in our experiment, total lipids were measured once the dynamics of the N:C ratio was stabilized (Fig. 1), i.e., 6 days after the beginning of the starvation. After several days of starvation, important variations in lipid accumulation are expected as a consequence of the decrease in the N:C ratio [15]. Unfortunately, very few authors have measured the N:C ratio simultaneously with the lipid content. Without the cell nitrogen quota, the actual cell nitrogen status stays unclear and comparing studies with potentially different physiological starvation state is risky.

### Lipid accumulation under low nitrogen limitations

Under moderate N-limited conditions, strategies to store carbon strongly differ between species. In *D. salina*, the triglyceride content was negatively correlated to the N:C ratio, as in *Neochloris oleoabundans* [38]. For other species such as *Isochrysis affinis galbana*, the relation is less clear, i.e., either positive or non-significant correlation [15, 39].

In nitrogen-replete conditions, the triglyceride content of *D. Salina* reached its lower level (0.016 ± 0.004 µg µgC^−1^), in the range found by [24]. This minimal triglyceride quota (5% of total lipids in *D. salina*) is frequently reported in the literature [38, 39], and represents the minimal needs in terms of triglycerides for cells in non-stressing conditions. Some studies showed that triglycerides are not only a final product in lipid anabolism but also constitute intermediary products in biosynthesis of glycolipids and phospholipids [42]. Moreover, the idea that lipid droplets, mainly containing triglycerides, are more than simple storage organelles but are inter-connected with other functional roles is emerging, that might corroborate the need for a minimal triglyceride quota [43–45].

### Polar lipids versus nitrogen status

A sigmoidal response of polar lipids was observed as a response to nitrogen limitation. The minimum level reached in N-starved cells represents 30% of total lipids (0.12 ± 0.004 µg µgC^−1^) and corresponds to structural lipids mainly dedicated to cell and organelle membranes. In N-replete conditions, the range of polar lipids (from 0.2 to 0.3 µg µgC^−1^) is consistent with the results reported by [24].

### Concomitant changes of triglycerides and beta-carotene

Beta-carotene has three major functions (a) to absorb excess of light to avoid damage of the photosynthetic machinery, (b) to resorb reactive oxygen species created by the production of oxygen under high photon flux, (c) to be a carbon sink when cells receive excess energy in unbalanced growth. Under nitrogen limitation, growth is reduced, and excess energy and carbon from photosynthesis are stored into molecules which do not contain nitrogen: beta-carotene and triglycerides [11]. Triglycerides are accumulated into lipid droplets delimited by a phospholipid monolayer, stabilized by carotene globule proteins [10]. In *D. salina*, these lipid droplets also contain beta-carotene [8]. The range of beta-carotene content (10–32 g L^−1^ of biovolume) was in accordance with the literature (between 2 and 15 g L^−1^ of biovolume; [46]) for the same strain CCAP 19/18. Very few studies have explored the link between triglycerides and beta-carotene. Rabbani et al. [8] showed that an inhibition of the triglycerides pathway also inhibits beta-carotene accumulation. They concluded a causal interdependence between lipid droplet formation and the increase in beta-carotene content. They hypothesized that beta-carotene synthesis was stimulated by the lipophilic sink created by triglyceride droplets (package effect).

Our work demonstrated for the first time the linear relation between these two molecules. Although *D. salina* lipid droplet is known to contain triglycerides and beta-carotene, our results reveal a higher content in beta-carotene than triglycerides. This is inferred from the slope of the linear relation found in Fig. 5 which was higher than 1 (1.32 µg_Beta-carotene_ µg_Triglycerides_^−1^ or 1.79 mol_Beta-carotene_ mol_Triglycerides_^−1^) and is in accordance with previous conclusions [12]. Borel et al. [47] showed that beta-carotene solubility in artificial triolein droplets stabilized by phospholipids had a maximum value of 0.3 µg_Beta-carotene_ µg_triolein_^−1^ much lower than the 1.32 µg_Beta-carotene_ µg_Triglycerides_^−1^. This unexpected overconcentration is possibly due to lipid droplet structure and the presence of proteins that stabilized the emulsion.

Under starvation, the beta-carotene and triglyceride content was uncoupled (Fig. 5). In this particular physiological state, starvation decreased the nitrogen quota and probably the carotenoid globule protein production. We hypothesize that at a certain threshold, there were not enough proteins to stabilize lipids droplets; triglycerides content continued to increase but the lipid droplet volumes remained constant. The triglycerides lipophilic sink effect demonstrated by [8] was slowed down, leading to a reduction of beta-carotene synthesis.

Mendoza et al. [12], demonstrated a linear correlation between beta-carotene and oleic acid in *D. salina*. They did not observe the late uncoupling probably because they worked in nitrogen-replete conditions and reach lower beta-carotene content (12 ρg cell^−1^ instead of 20.4 ρg cell^−1^ in our study).

## Conclusion

This study highlights the growth and lipid production of *D. salina* for different levels of nitrogen limitation. Storing carbon into lipids turns out to be species dependent, especially during nitrogen limitation. The strong correlation, in nitrogen limitation, between triglycerides and beta-carotene was confirmed, and the uncoupling due to nitrogen starvation was observed. Further studies should be carried out to better understand lipid droplet formation and structure and their role in beta-carotene production, especially in conditions of nitrogen starvation where the production of key stabilizing proteins might be reduced. The mechanism of beta-carotene storage into lipid droplets is still unclear and should be investigated at a proteomic level.

## Abbreviations

N replete, starvation or limitation: nitrogen replete, starvation or limitation
N:C ratio: nitrogen-to-carbon ratio of the biomass (g g^−1^)
*q*: amount of nitrogen per carbon unit (i.e., the N:C ratio)
*q*_0_: minimum value of *q* below which growth was not possible
*k*_s_: half saturation constant
*µ*: net carbon specific growth rate (µgC µgC^−1^ d^−1^)
$\bar{\mu }$: theoretical maximum growth rate obtained when *q* is assumed to be infinite
dw: dry weight
TLC: thin layer chromatography
*D*: dilution rate (d^−1^)
*X*: particulate carbon concentration (biomass µgC mL^−1^)
*s*: nitrate concentration in the culturing medium (µmol L^−1^)
*s*_0_: nitrate concentration in the input medium (µmol L^−1^)
*ρ*(*s*): uptake rate of the limiting nutrient per unit of *X*
*ρ*_m_: maximum uptake rate
